# Protocol for simulating macrophage signal transduction and phenotype polarization using a large-scale mechanistic computational model

**DOI:** 10.1016/j.xpro.2021.100739

**Published:** 2021-08-12

**Authors:** Chen Zhao, Aleksander S. Popel

**Affiliations:** 1 School of Pharmacy, Nanjing Medical University, Nanjing, Jiangsu 210000, China; 2 Department of Biomedical Engineering, Johns Hopkins University School of Medicine, Baltimore, MD 21205, USA

**Keywords:** Immunology, Signal Transduction, Systems biology, Computer sciences

## Abstract

The ability to measure and analyze the complex dynamic multi-marker features of macrophages is critical for the understanding of their diverse phenotypes and functions in health and disease. To that end, we have recently developed a multi-pathway computational model that for the first time enables a systems-level characterization of macrophage signaling and activation from quantitative, temporal, dose-dependent, and single-cell aspects. This protocol includes instructions to utilize this model to computationally explore different biological scenarios with high resolution and efficiency.

For complete details on the use and execution of this protocol, please refer to Zhao et al. (2021).

## Highlights

• A computational platform of multi-pathway mechanistic macrophage polarization
• Model resolution from quantitative, time/dose-dependent, and single-cell aspects
• Model-based evaluation of therapeutic macrophage repolarization in pathophysiology

## Graphical abstract

**Figure:**
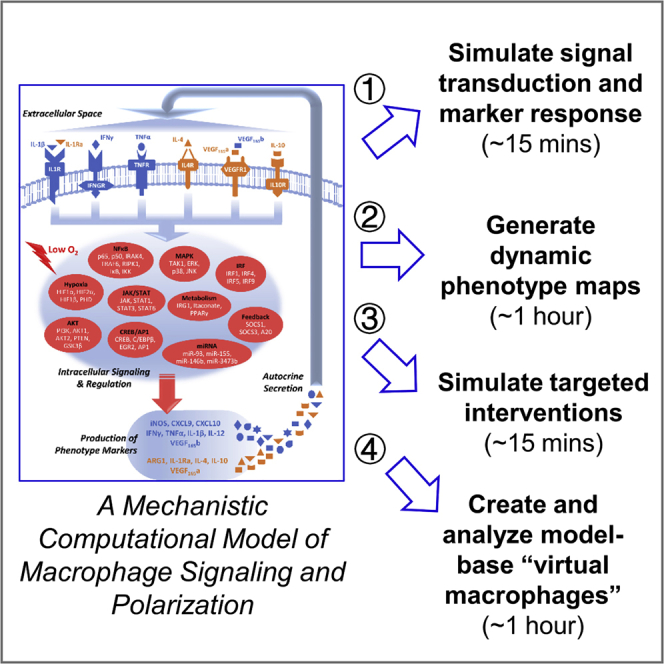

## Before you begin

The computational model presented here, as a simulation tool, is a significant step toward the future formulation and development of a comprehensive network-centric “virtual macrophage” *in silico* platform. This computational model takes inputs which are quantitative changes in the initial conditions of certain modeled species (e.g., ligands); then the model is simulated for a user-specified duration (using minutes as the smallest time unit) to produce time-dependent, numerical outputs (in terms of the quantitative levels of all modeled species). Compared to other published models with similar focus, our model has achieved a much higher level of model performance accuracy given the extensive amount of calibration and validation implemented (Zhao et al., 2021, Liu et al., 2021, Zhao et al., 2019, Rex et al., 2016, Maiti et al., 2015). This model is formulated based on ordinary differential equations (ODEs) and is implemented in MATLAB (MathWorks, Natick, MA); thus, syntax checks and changes may be required to run the model SBML code in another programming language, e.g., Python.

In this protocol, we describe examples that used our model to simulate macrophage pathway signal transduction, generate dynamic polarization maps, perform *in silico* intervention experiments, and create a model-based population of “virtual single cells”. These examples are demonstrated using scripts in MATLAB (a 2016 release or later version is recommended); the Simbiology toolbox in MATLAB is required.

### Initial setup and basic framework of the computational model

**Timing: about 30 mins**

1. Download the model file package (.zip file) from https://github.com/czhaoqsp/mac_sig_model; extract the files from the package.
  a. Open MATLAB software.
  b. In MATLAB, click “Browse for folder” in the “Current Folder” window and open the folder containing the model files.
2. To load the model and see its basics, type “m = sbmlimport('7pathmodel_clean_v2.xml');” in the command window and then press the Enter key. Make sure the model name is entered correctly (Troubleshooting 1).
  a. To see the overall model features, type “m” in the command window and then press Enter.
  b. To see the description of all model parameters, type “m.Parameters” in the command window and press Enter. To see the description of a specific parameter (e.g., parameter #1), type “m.Parameters(1)” and then press Enter.
  c. To see the description of all modeled species, type “m.Species” in the command window and press Enter. To see the description of a specific species (e.g., species #1), type “m.Species(1)” and then press Enter.
  d. To see the description of all model reactions, type “m.Reactions” in the command window and press Enter. To see the description of a specific reaction (e.g., reaction #1), type “m.Reactions(1)” and then press Enter.

## Key resources table

**Table:** 

REAGENT or RESOURCE	SOURCE	IDENTIFIER
**Software and algorithms**		

Executable model code and scripts	(Zhao et al., 2021)	https://github.com/czhaoqsp/mac_sig_model

**Other**		

Excel files listing all model reactions, species, and parameters	(Zhao et al., 2021)	https://doi.org/10.1016/j.isci.2021.102112
Excel files listing all quantitative data used in model calibration and validation (compare to model simulations)	(Zhao et al., 2021)	https://doi.org/10.1016/j.isci.2021.102112
MATLAB (and Simbiology toolbox)	https://www.mathworks.com/products/matlab.html	https://www.mathworks.com/products/matlab.html

## Step-by-step method details

### Simulating the effect of ligand stimulations

**Timing: about 15 mins**

In this section, we will show how to use the model to simulate the downstream signal transduction of driving pathway stimulations.

1. In the “Current Folder” window in MATLAB, double click to open the file named “simulatemodel_samplescript.m”.
  a. Run the first section named “model setup and simulation” in the script. This will load the model.
  b. Run the second section named “simulation scenarios” in the script. This part contains two examples: hypoxia and IL-4 stimulation. The sample code will produce (e.g., plot) time-course profiles of cellular HIF1α (hypoxia inducible factor 1 alpha) in response to hypoxia, and ARG1 (arginase 1) in response to IL-4 stimulation.
    i. For the simulation of hypoxia, the initial condition of oxygen (O2), as the only input, was changed. This was reflected as numerical changes in the “InitialAmount” field of species #78.
    ii. For the simulation of IL-4 addition, the initial condition of IL-4, as the only input, was changed. This was reflected as numerical changes in the “InitialAmount” field of species #28.
    iii. To perform model simulations, a simulation timespan also needs to be set. This is achieved by the command “set(cs, 'StopTime', 1500)” where 1500 is the simulation timespan 1500 min. When specifying the simulation timespan, the configuration handler should be already obtained by the command “cs = getconfigset(m, 'active')”.
    iv. To set the ODE solver for the model, use command “cs.SolverType='ode15s'”. MATLAB provides many different ODE solvers; ode15s is the recommended solver for our model (Troubleshooting 2).
    v. To perform model simulations, the command is “[t,out] = sbiosimulate(m)”. Here, “t” is the vector containing all time points, “out” is the matrix containing all model outputs (of all modeled species).
2. To change the initial condition of specific modeled species, make sure to first identify the number of that particular species: use the “m.Species” command, and also refer to supplemental Tables S1 and S2 in (Zhao et al., 2021) for more details regarding species naming. Then change the ‘InitialAmount’ field of that particular species to the desired value (see above examples of oxygen and IL-4).
3. To obtain the specific model output of interest, make sure to first identify the number of that particular species before getting its value. For example, ARG1 (arginase 1) is #53 in all species, so the command “a1=out(:,53);” was used here to obtain the time-course values of ARG1.
4. To simulate the effect of multiple stimulations in different combinations and orders, follow the above instructions to change the species initial conditions accordingly.
5. When plotting the results, especially when plotting the time-course profiles of multiple species in one figure, data normalization is recommended for better visualization (e.g., species values are normalized to their respective maxima).

**CRITICAL:** Users should note that the default simulation output variable is a two-dimensional matrix, and each column in the matrix, instead of the row, is the time-course value of a species.

### Generation of dynamic macrophage polarization maps

**Timing:about 1 h**

In this section, we will show how to use the computational model to create a macrophage polarization map that shows diverse stimulation-induced phenotypes. Here we evaluated 28 different stimulation conditions (that are combinatorially derived from the 7 driving pathways described by the model).

6. In the “Current Folder” window in MATLAB, double click to open the file named “analysis_Fig5_polarizationmap.m”.
  a. Run the first section of the script. Here, a total number of 3x28 stimulation conditions are simulated (3 time points; 7 single stimulus plus 21 pairwise combinations). This will generate the source data for the polarization map (in lines 3456-3458, variables sum4vitro, sum24vitro and sum48vitro contain relative fold changes of a panel of macrophage markers evaluated at 4, 24 and 48 h, respectively). With these data, the polarization maps can then be created and visualized in MATLAB using the function *heatmap* (Troubleshooting 3).
  b. Run the second section of the script (starting at line 3463). This will plot time-course trends (evaluated at 4, 24 and 48 h) of the relative M1/M2 scores of 7 single stimulus and 21 pairwise stimulus conditions. The results are shown in log scale for better comparison. Details of the M1/M2 score are discussed in (Zhao et al., 2021).

### Simulating intervention experiments *in silico*

**Timing:about 15 mins**

In this section, we will show how to use the computational model to simulate the effect of certain targeted interventions on macrophage phenotypes. Examples here used hypoxia (2 percent oxygen) as the control condition.

7. In the “Current Folder” window in MATLAB, double click to open the file named “analysis_Fig6_targetedintervention.m”.
8. Run the first section of the script. This will load the model.
9. Run the third section of the script (starting at line 94). In this example, we compared the macrophage phenotype upon SOCS1 inhibition (by accelerated degradation) in hypoxia to the phenotype in the control condition. This targeted intervention (inhibiting SOCS1 by promoting its degradation) was achieved by increasing the protein degradation rate of SOCS1 by 10 fold in the model (using the command “m.Parameters(125).Value=0.004∗10;”).
10. Usually, different kinds of targeted interventions (e.g., protein overexpression and inhibition, inhibition of a protein-protein interaction process) can be accordingly simulated as changes in the species initial conditions or parameter values.

### Creation and analysis of model-based virtual single cells

**Timing:about 1 h**

In this section, we will show how to use the computational model to create and analyze a population of virtual single cells (macrophages).

11. In the “Current Folder” window in MATLAB, double click to open the file named “analysis_Fig7_virtualmacs.m”.
12. Run the first section of the script. This will load the model.
13. Run the second section of the script. This will create a sample model-based population of 100 virtual single cells.
  a. Here, we selected a set of 42 parameters that control the production and degradation of 10 modeled species (5 are important intracellular regulators, 5 are cytokines) and reassigned new values to them. This procedure was done 100 times to generate 100 new cells (in terms of 100 new parameterization of the set of 42 parameters). More technical details can be found in Transparent Methods in (Zhao et al., 2021).
14. Run the third section of the script (starting at line 163). This will plot the time-dependent M1-M2 phenotypes of the 100 newly generated virtual macrophages in hypoxia, as 100 trajectories (in terms of their relative M1 scores, M2 scores, and M1/M2 scores) (Troubleshooting 5).
  a. The phenotype behaviors of the original reference model were also plotted (in black bolded line).
15. Run the fourth section of the script (starting at line 253). This will plot the time-dependent M1-M2 phenotypes of the 100 virtual macrophages in response to hypoxia plus interventions targeting STAT3 or STAT6, as 100 trajectories (in terms of their relative M1/M2 scores).
  a. Interventions targeting STAT3 or STAT6 were simulated here as decrease in their dephosphorylation rates (by changing the corresponding parameter values).
  b. The phenotype behaviors of the original reference model were also plotted here to enable comparison (in black bolded line).

## Expected outcomes

**Figure 1:**
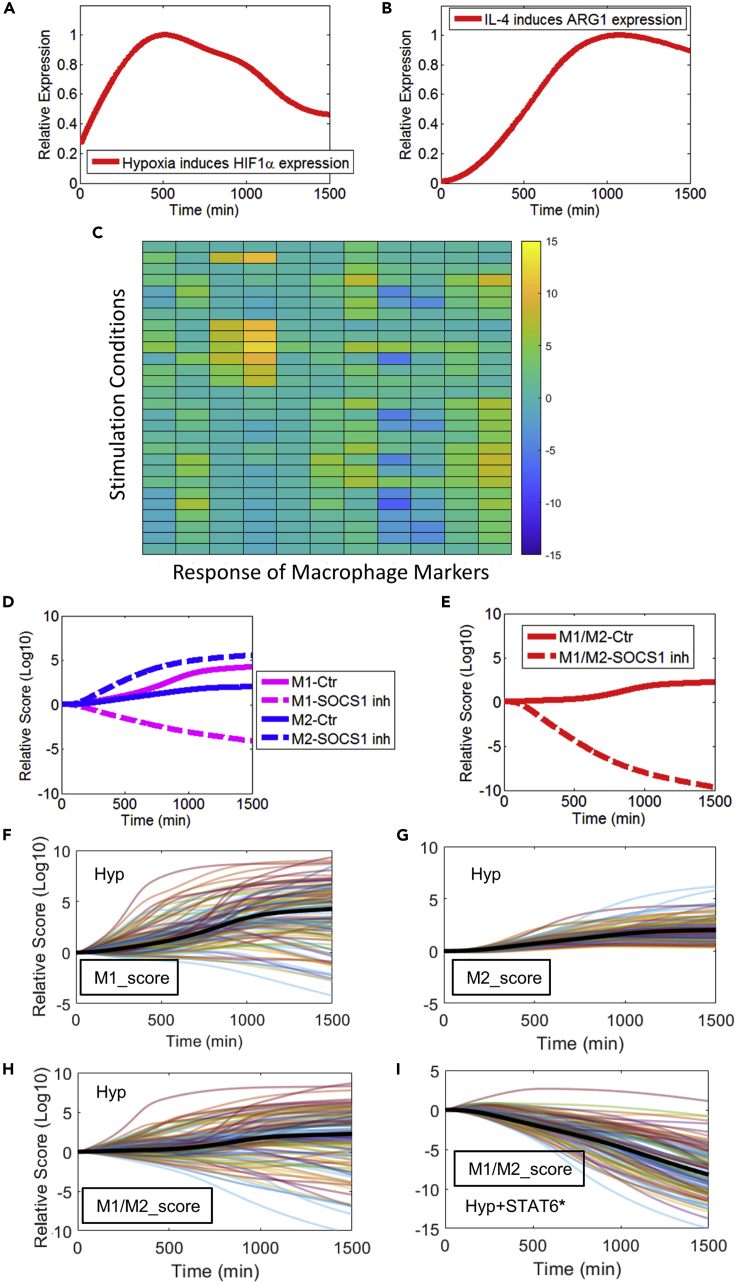
Expected outcomes of simulation examples discussed in this protocol (A and B) Steps 1–5: simulated time-course expression profiles of HIF1α (upon hypoxia) and ARG1 (upon IL-4 stimulation). Simulation results are normalized to the respective maximum values. (C) Step 6: an example of a simulated macrophage phenotype map. Here, the values for the relative fold changes of all markers are taken at 4 h and then log2 transformed. More details of this map can be found in the MATLAB script provided. (D and E) Steps 7–10: simulated phenotype profiles (in terms of M1, M2, and M1/M2 scores) of macrophages in hypoxia versus hypoxia plus SOCS1 inhibition. (F–H) Steps 11–14: simulated phenotype profiles (in terms of relative M1, M2, and M1/M2 scores) of 100 model-based “virtual macrophages” in hypoxia (Hyp). (I) Step 15: simulated phenotype profiles (in terms of the relative M1/M2 scores) of 100 model-based “virtual macrophages” in response to hypoxia and an *in silico* intervention targeting STAT6. (D–I) Results are normalized to their respective t=0 values and then log10 transformed.

In Figure 1, we illustrate the expected outcomes of the simulation examples provided in this protocol. For simulation of macrophage signal transduction and marker expression, Figures 1A and 1B show the simulated intracellular expression of HIF1α in response to hypoxia, and expression of ARG1 in response to IL-4 ligand stimulation. For generation of macrophage phenotype maps, Figure 1C shows the simulated diverse response of a multi-marker panel under an array of stimulation conditions. For simulation of targeted intervention, Figures 1D and 1E show the relative macrophage phenotype scores (M1, M2, and M1/M2) in hypoxia versus hypoxia plus SOCS1 inhibition. For analysis of virtual single cells, Figures 1F–1H show the phenotype trajectories of 100 virtual macrophages (in terms of their relative M1, M2, and M1/M2 scores) in hypoxia; Figure 1I show the phenotype trajectories of the same 100 virtual macrophages (in terms of their relative M1/M2 scores) in response to hypoxia plus a simulated intervention that increases STAT6 activation. For a more complete description of model results, please refer to the content in (Zhao et al., 2021).

## Limitations

We anticipate our computational model to be further expanded and improved in future to more mechanistically and accurately deliver the idea of building a comprehensive, simulatable virtual macrophage. Here we describe several limitations associated with our model and its applications. First, the current model version, as detailed here and in (Zhao et al., 2021), includes only 7 driving pathways, thus simulations of pathway signal transduction and phenotype polarization maps are restricted to combinations of these 7 pathways. Second, at the *in vitro* level, the model did not explicitly consider the potential influence of different cell plating densities on the experimental outcomes (Lee and Hu, 2013). Third, when generating dynamic macrophage polarization maps, the current protocol explored only three discrete time points; still, the model can be used to simulate macrophage phenotypes at any particular time point. Fourth, in this protocol, the method we used to calculate M1/M2 score (which considers the relative fold changes of 11 canonical M1 and M2 markers) is only one of the many ways that researchers can use to define macrophage phenotypes, and it should be adapted (e.g., consider different marker combinations) for different simulation scenarios and study purposes. More discussion regarding limitations can be found in (Zhao et al., 2021).

## Troubleshooting

### Problem 1

Model file not found or cannot be loaded (Initial Setup).

### Potential solution

Make sure the folder that directly contains the model SBML (.xml) file is currently opened in MATLAB “Current Folder” window. Also make sure that the name of the xml file is exactly the same as it appears in the *sbmlimport* command.

### Problem 2

Model simulation settings cannot be specified (step 1).

### Potential solution

Make sure that the handler for the model configuration has been obtained using the *getconfigset* command before specifying other simulation settings such as simulation timespan and solver type.

### Problem 3

Heatmap cannot be created (step 6).

### Potential solution

To create heatmaps in MATLAB, the function to use is slightly different in different MATLAB versions in terms of capitalization (e.g., *HeatMap* for R2014a, *heatmap* for R2018a). Please check the help files in the exact MATLAB version used.

### Problem 4

The simulation result does not look reasonable (all steps).

### Potential solution

Although this problem may be caused by many different reasons, we would like to point to one mistake that could be quite likely committed during coding. When assigning new initial conditions to species and new values to parameters, the changes are permanent to the current model variable/instance loaded. Thus, when a new condition is to be simulated, the user needs to make sure that the previously modified initial conditions and parameter values have been restored to their original values. An alternative way is to load a new model instance with the same name to overwrite the previous model instance.

### Problem 5

The for loop terminated prematurely due to simulation errors (steps 14 and 15).

### Potential solution

Given that the resampled parameter sets used here have a random component, it is possible (although very rarely) that some simulations can run into integration errors in MATLAB. To detect this and prevent the for loop from terminating prematurely (especially when simulating a large number of individual cells), users can add the *try/catch* statements here (see https://www.mathworks.com/help/matlab/ref/try.html for more details about *try/catch*).

## Resource availability

### Lead contact

Further information and requests for resources and instructions should be directed to and will be fulfilled by the lead contact, Dr. Chen Zhao (zcshinchon4677@outlook.com).

### Materials availability

This study did not generate new unique reagents.

## Data Availability

Details of all model reactions, equations, parameters, initial conditions, the complete model coded in SBML format (.xml file) and executable MATLAB scripts (.m files) that can run the model to generate sample simulations are provided in https://doi.org/10.1016/j.isci.2021.102112.
